# Poor prognosis in stage III colorectal cancer with apical lymph node metastasis: a single-center retrospective study

**DOI:** 10.1007/s13304-025-02219-9

**Published:** 2025-05-05

**Authors:** Burak Dinçer, Ahmet Karayiğit, Fatma Markoç, Serdar Sarıdemir, Cihangir Özaslan

**Affiliations:** 1 Department of Surgical Oncology, University of Health Sciences, Gülhane School of Medicine, Ankara Oncology Training and Research Hospital, Mehmet Akif Ersoy Mah. Vatan Cad. No: 91 Yenimahalle, Ankara, Türkiye; 2 Department of Pathology, University of Health Sciences, Gülhane School of Medicine, Ankara Oncology Training and Research Hospital, Ankara, Türkiye

**Keywords:** Apical lymph node, Colorectal cancer, Metastasis, TNM staging, JCCRC staging

## Abstract

The impact of apical lymph node (ALN) status on the prognosis of colorectal cancer (CRC) remains controversial, and ALN status is not included in the current Tumor Node Metastasis (TNM) staging system. This study aimed to evaluate the effect of ALN status on recurrence and survival rates. In this retrospective study, 117 stage 3 CRC patients aged over 18 who underwent surgery between 2015 and 2024 and had their ALN status determined were evaluated. Patients with metastatic disease at diagnosis, those with undetermined ALN status, and those with concurrent malignancies were excluded. Patients were analyzed based on demographic, clinical, pathological, and survival data. The median age was 61 years (range: 33–83), and 60.7% of the patients were male. The pN stage was significantly more advanced (*p* < 0.001) and the number of metastatic lymph nodes was significantly higher (*p* = 0.003) in the ALN ( +) group. During a median follow-up of 46 months, 14 local recurrences, 31 systemic recurrences, and 27 cancer-related deaths were observed. Local recurrence, systemic recurrence, and cancer-related deaths were significantly more frequent in the ALN ( +) group (*p* = 0.027, *p* < 0.001, and *p* < 0.001, respectively). Locoregional disease-free survival, systemic disease-free survival and overall survival were significantly shorter in the ALN ( +) group (*p* = 0.011, *p* < 0.001, and *p* < 0.001, respectively). In multivariate analysis, SDFS and OS were found to be significantly shorter in the ALN ( +) and pN2 groups. ALN metastasis can be considered as an additional adverse prognostic factor in CRC beyond the pN stage.

## Introduction

Colorectal cancer (CRC) is the third most common cancer and the second leading cause of cancer-related mortality, according to the World Health Organization's 2022 data [1]. Since CRC is prevalent and has a curative treatment option when diagnosed at an early stage, many countries have included CRC in their screening programs [2]. Despite these programs, a significant proportion of patients are still diagnosed at an advanced stage, with synchronous systemic metastases detected in 20–30% of newly diagnosed cases[3]. The most common metastasis is mesenteric lymph node involvement, which is considered a poor prognostic indicator.

Several staging systems have been developed to predict prognosis in CRC and guide appropriate adjuvant treatment selection. The American Joint Committee on Cancer (AJCC) Tumor-Node-Metastasis (TNM) staging system is the most widely used worldwide. In this system, lymphatic staging is based on the number of metastatic lymph nodes, whereas the localization of metastatic lymph nodes is not incorporated into the staging criteria [4]. In contrast, the Japanese Classification of Colorectal, Appendiceal, and Anal Carcinoma (JCCRC) staging system considers both the number and localization of metastatic lymph nodes, categorizing apical lymph node (ALN) metastasis as N3 [5]. Although the N3 category was once included in the TNM classification, it was later abandoned due to ongoing controversy regarding the prognostic significance of ALN involvement [6, 7].

In addition to ALN status, other prognostic factors, such as the metastatic/total lymph node ratio and the presence of tumor deposits, have been investigated in the literature, yielding controversial results [8–15]. This study aimed to analyze stage III CRC patients who underwent surgery at our center and to evaluate the impact of ALN involvement on recurrence rates and survival outcomes in this patient population.

## Materials and methods

### Study population and selection criteria

**Fig. 1:**
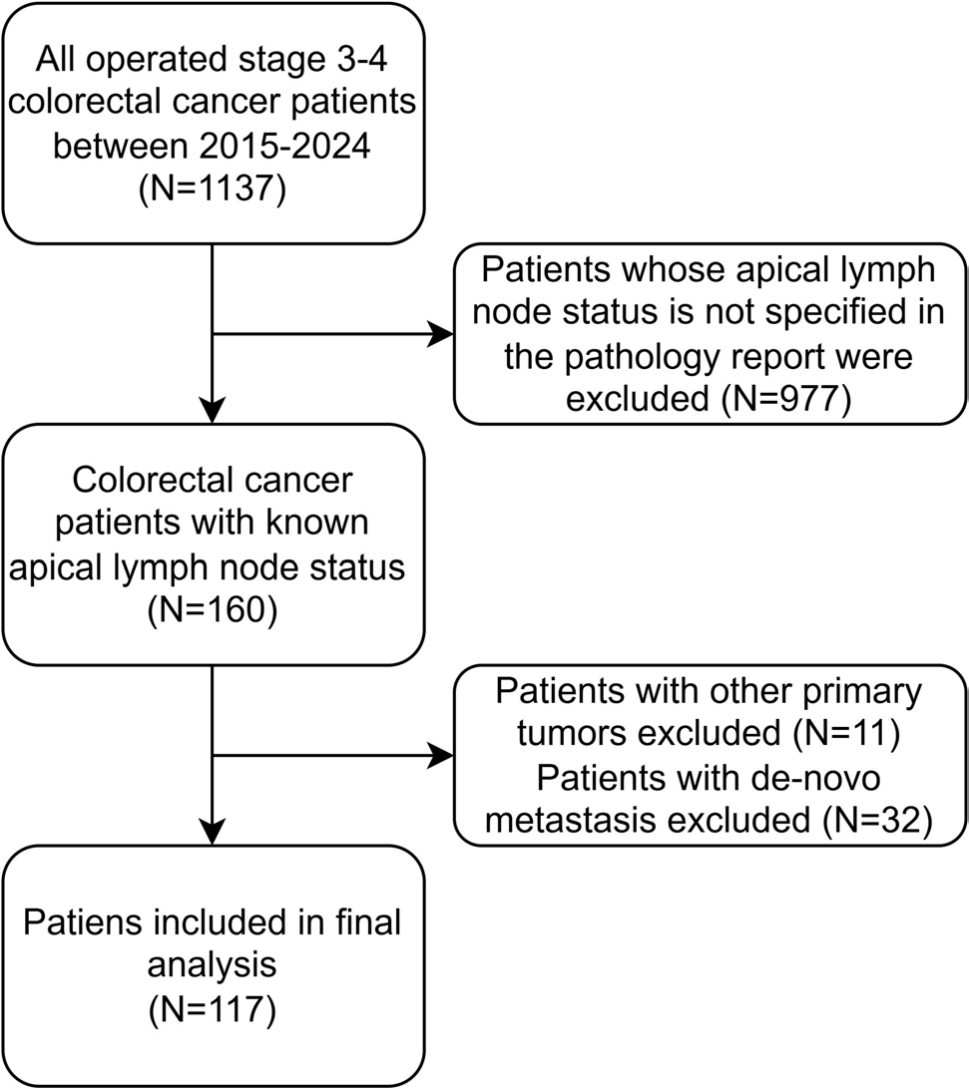
Flowchart of study participants

A retrospective review was conducted on 1,134 stage III–IV CRC patients who underwent surgery at our center between 2015 and 2024. Patients with metastatic disease at the time of diagnosis, those with concurrent malignancies, and those with undetermined ALN status were excluded. Ultimately, 117 patients with stage III CRC and known ALN status were included in the final analysis **(**Fig. 1**)**.

This study was conducted in accordance with the Declaration of Helsinki and was approved by the local ethics committee (October 31, 2024—No: 2024–10/153). Patients aged ≥ 18 years who underwent surgery for CRC were included. Exclusion criteria included patients with unspecified ALN status in the pathology report, systemic metastasis at diagnosis, concurrent malignancies, and pN0 status.

Patients were evaluated based on age, sex, American Society of Anesthesiologists (ASA) physical status score [16], neoadjuvant and adjuvant treatments, surgical technique, complications, postoperative mortality, pathological diagnosis, tumor differentiation, pT and pN stage, surgical margin status, ALN status, recurrence rates, and survival outcomes.

Survival time was defined as the duration from the date of surgery to the occurrence of the determined event. Locoregional disease-free survival (LDFS) was defined as the time until the development of any locoregional recurrence, systemic disease-free survival (SDFS) as the time until the occurrence of systemic metastasis, and overall survival (OS) as the time until death. For patients without an event, survival was defined as the time from surgery to the last follow-up.

### Surgical techniques

In patients diagnosed with CRC, treatment selection is based on tumor location. At our center, a complete mesocolic approach is routinely performed for lymphatic dissection, and central (apical) lymph node areas are dissected (D3 dissection) according to tumor location. Additionally, relevant vascular structures are ligated at their origins using a high-ligation technique. The surgical approach varies depending on tumor location: right hemicolectomy for ascending colon tumors, extended right or left hemicolectomy or transverse colectomy for transverse colon tumors, left hemicolectomy for descending colon tumors, anterior resection for sigmoid tumors, and either low anterior resection or abdominoperineal amputation for rectal tumors. While a laparoscopic approach is preferred in suitable cases, an open technique may be chosen for locally advanced tumors or patients with a history of prior abdominal surgery.

### Pathological analysis of apical lymph nodes

For right hemicolectomy, lymph nodes at the origins of the ileocolic artery and vein and the right colic artery and vein were classified as ALN. For transverse colectomy, lymph nodes at the origin of the middle colic artery and vein were considered ALN. For left hemicolectomy, anterior resection, low anterior resection, and abdominoperineal amputation, lymph nodes at the origins of the inferior mesenteric artery and vein were classified as ALN. Pericolic lymph nodes were defined as those surrounding the colon, while intermediate lymph nodes were those located between the pericolic and apical regions.

Lymph nodes obtained from approximately 1–2 cm of fatty tissue at the origin of the relevant vascular structure in the pathology specimen were considered as ALN.

### Adjuvant treatments and follow-up

Neoadjuvant chemoradiotherapy (CRT) was administered to patients with locally advanced rectal cancer routinely. In patients with locally advanced colon cancer, upfront surgery is generally preferred; however, neoadjuvant chemotherapy may also be administered. Following completion of wound healing after surgery, all patients were evaluated by the medical oncology unit for adjuvant treatment. All stage III patients with adequate performance status to tolerate treatment received adjuvant chemotherapy (CT). Capecitabine- and oxaliplatin-based regimens were generally administered. All patients included in the study successfully completed their adjuvant CT protocols.

Patients were followed up every 3 months for the first 2 years, every 6 months for the next 3 years, and annually thereafter. Routine follow-up included physical examination (including digital rectal examination) and tumor marker assessment. Systemic imaging, including thoracic and abdominal computed tomography or, if necessary, positron emission tomography, was performed at 6-month intervals. Colonoscopic evaluation was conducted 6–12 months after surgery, with subsequent surveillance colonoscopies scheduled based on initial findings.

For patients with local recurrence, surgery was planned if resection was feasible. Among patients who developed systemic metastases, surgery was considered for those with resectable liver or lung metastases. Chemotherapy was administered to all patients with recurrence who had sufficient performance status, regardless of surgical candidacy.

### Outcome measures

The impact of ALN metastasis on local and systemic recurrence rates, LDFS, SDFS and OS was evaluated as the primary outcome. The prevalence of ALN metastasis in the entire cohort was assessed as the secondary outcome.

### Statistical analysis

Statistical analysis was performed using SPSS® (Statistical Package for the Social Sciences) version 25.0 (IBM Corp., Armonk, NY, USA). Descriptive statistics, including count, percentage, and median, were used to summarize the study data. The normality of continuous variables was assessed using the Shapiro–Wilk test. Normally distributed continuous variables were presented as mean ± standard deviation, while non-normally distributed variables were expressed as median and interquartile range (IQR). Survival data were analyzed using Kaplan–Meier curves, and factors affecting survival were assessed with the log-rank test. Categorical variables were analyzed using Chi-square tests (Pearson Chi-square, Fisher's Exact Test), while continuous variables were compared using Student's t-test or the Mann–Whitney U test. A p-value of < 0.05 was considered statistically significant, with a 95% confidence interval.

## Results

The median age was 61 years (range: 33–83), and 60.7% of the 117 patients included in the study were male. A total of 60.7% of the patients were classified as ASA score 2. The most common tumor location was the rectum (58.1%), followed by the sigmoid colon (30.8%). Neoadjuvant CT was administered to 52 patients, while 54 patients received neoadjuvant RT. Adjuvant CT was given to 111 patients, and adjuvant RT was administered to 25 patients. No significant differences were observed between ALN (−) and ALN (+) patients in terms of demographic and clinical parameters. (Table 1).

**Table 1:** Demographic and clinical characteristics of the study group

Variables	All patients (N = 117)	ALN (-) Patients (N = 99)	ALN (+) Patients (N = 18)	P-value
Age (Years, Median, Range)	61 (33–83)	61 (33–83)	64 (33–74)	*0.904*^*a*^
Sex (N, %)				
Female	46 (39.3%)	36 (30.8%)	10 (8.5%)	*0.125*^*b*^
Male	71 (60.7%)	63 (53.8%)	8 (6.8%)	
ASA score (N, %)				
ASA 1	2 (1.7%)	1 (0.9%)	1 (0.9%)	*0.364*^*c*^
ASA 2	71 (60.7%)	61 (52.1%)	10 (8.5%)	
ASA 3	44 (37.6%)	37 (31.6%)	7 (6%)	
Tumor Localization (N, %)				
Ascending colon	5 (4.3%)	4 (3.4%)	1 (0.9%)	*0.297*^*c*^
Transverse colon	1 (0.9%)	0 (0%)	1 (0.9%)	
Descending colon	7 (6%)	6 (5.1%)	1 (0.9%)	
Sigmoid colon	36 (30.8%)	30 (25.6%)	6 (5.1%)	
Rectum	68 (58.1%)	59 (50.4%)	9 (7.7%)	
Neoadjuvant CT (N, %)				
No	65 (55.6%)	55 (47%)	10 (8.5%)	*1.000*^*b*^
Yes	52 (44.4%)	44 (37.6%)	8 (6.8%)	
Neoadjuvant RT (N, %)				
No	63 (53.8%)	53 (45.3%)	10 (8.5%)	*0.874*^*b*^
Yes	54 (46.2%)	46 (39.3%)	8 (6.8%)	
Adjuvant CT (N, %)				
No	6 (5.1%)	6 (5.1%)	0 (0%)	*0.588*^*c*^
Yes	111 (94.9%)	93 (79.5%)	18 (15.4%)	
Adjuvant RT (N, %)				
No	92 (78.6%)	78 (66.7%)	14 (12%)	*1.000*^*c*^
Yes	25 (21.4%)	21 (17.9%)	4 (3.4%)	

*ALN* apical lymph node, *ASA* American society of anesthesiologists, *CT* chemotherapy, *RT* radiotherapy

^a^Mann–Whitney *U*-test, ^b^Chi-square test, ^c^Fisher’s exact test

The most frequently performed surgical procedure was low anterior resection (41.9%). Laparoscopic surgery was performed in 64 patients. A total of 23 patients developed postoperative complications, 15 of which were classified as major complications (Clavien-Dindo Grade ≥ 3). Postoperative mortality within the first 30 days was observed in two patients. No significant differences were found between ALN (−) and ALN (+) patients regarding surgery-related parameters. (Table 2).

**Table 2:** Surgical parameters of the study population

Variables	All patients (N = 117)	ALN (−) Patients (N = 99)	ALN (+) Patients (N = 18)	P-value
Surgical Technique (N, %)				
Right Hemicolectomy	6 (5.1%)	4 (3.4%)	2 (1.7%)	*0.746*^*a*^
Left Hemicolectomy	6 (5.1%)	5 (4.3%)	1 (0.9%)	
Anterior Resection	35 (29.9%)	29 (24.8%)	6 (5.1%)	
Low Anterior Resection	49 (41.9%)	43 (36.8%)	6 (5.1%)	
Abdominoperineal Amputation	19 (16.2%)	16 (13.7%)	3 (2.6%)	
Total Colectomy	2 (1.7%)	2 (1.7%)	0 (0%)	
Laparoscopic Surgery (N, %)				
No	53 (45.3%)	44 (37.6%)	9 (7.7%)	*0.663*^*b*^
Yes	64 (54.7%)	55 (47%)	9 (7.7%)	
Complications (N, %)				
No complication	92 (78.6%)	78 (66.7%)	14 (12%)	*0.833*^*a*^
Grade 1	3 (2.6%)	3 (2.6%)	0 (0%)	
Grade 2	5 (4.3%)	4 (3.4%)	1 (0.9%)	
Grade 3a	0 (0%)	0 (0%)	0 (0%)	
Grade 3b	11 (9.4%)	9 (7.7%)	2 (1.7%)	
Grade 4a	4 (3.4%)	3 (2.6%)	1 (0.9%)	
Grade 5 (Postoperative mortality)	2 (1.7%)	2 (1.7%)	0 (0%)	
First 30-day Postoperative Mortality (N, %)				
No	115 (98.3%)	97 (82.9%)	18 (15.4%)	*1.000*^*a*^
Yes	2 (1.7%)	2 (1.7%)	0 (0%)	

*ALN* apical lymph node

^a^Fisher’s exact test, ^b^Chi-square test

Regarding pathological findings, 105 patients were diagnosed with adenocarcinoma, and moderate differentiation was observed in 85 cases. The most common pT stage was pT3, observed in 84 patients. Among pN stages, pN1a was the most frequent, with 38 patients classified as pN1a. Surgical margin positivity was detected in nine patients, all of whom had radial margin involvement. The median total number of lymph nodes examined was 16 (IQR: 14–21), with a median of 2 metastatic lymph nodes (IQR: 1–4). The median number of apical lymph nodes was 1 (IQR: 1–2). The ALN (+) group had a significantly higher pN stage (p < 0.001) and a greater number of metastatic lymph nodes (*p* = 0.003) compared to the ALN (-) group, while other pathological parameters were similar between the two groups. **(**Table 3**).**

**Table 3:** Pathological parameters of the study population

Variables	All patients (N = 117)	ALN (-) Patients (N = 99)	ALN (+) Patients (N = 18)	P-value
Pathological Diagnosis (N, %)				
Adenocarcinoma	105 (89.7%)	89 (76.1%)	16 (13.7%)	*1.000*^*a*^
Mucinous Adenocarcinoma	12 (10.3%)	10 (8.5%)	2 (1.7%)	
Degree of Differentiation (N, %)				
Well	26 (22.2%)	24 (20.5%)	2 (1.7%)	*0.504*^*a*^
Moderate	85 (72.6%)	70 (59.8%)	15 (12.8%)	
Poor	6 (5.1%)	5 (4.3%)	1 (0.9%)	
pT stage (N, %)				
T1	3 (2.6%)	3 (2.6%)	0 (0%)	*0.182*^*a*^
T2	11 (9.4%)	11 (9.4%)	0 (0%)	
T3	84 (71.8%)	71 (60.7%)	13 (11.1%)	
T4a	15 (12.8%)	12 (10.3%)	3 (2.6%)	
T4b	4 (3.4%)	2 (1.7%)	2 (1.7%)	
pN stage (N, %)				
N1a	38 (32.5%)	35 (29.9%)	3 (2.6%)	** < *0.001***^***a***^
N1b	33 (28.2%)	27 (23.1%)	6 (5.1%)	
N1c	12 (10.3%)	12 (10.3%)	0 (0%)	
N2a	22 (18.8%)	21 (17.9%)	1 (0.9%)	
N2b	12 (10.3%)	4 (3.4%)	8 (6.8%)	
Surgical margins (N, %)				
Negative	108 (92.3%)	92 (78.6%)	16 (13.7%)	*0.627*^*a*^
Positive	9 (7.7%)	7 (6%)	2 (1.7%)	
Total LN Count (Median, IQR)	16 (14–21)	16 (14–21)	19 (15–26)	*0.195*^*b*^
Metastatic LN Count (Median, IQR)	2 (1–4)	2 (1–4)	4 (2–8)	***0.003***^***b***^
Apical LN Count (Median, IQR)	1 (1–2)	1 (1–2)	1 (1–1)	*0.213*^*b*^
Metastatic Apical LN Count (Median, IQR)	0 (0–0)	0 (0–0)	1 (1–1)	*N/A*

All p-values less than 0.05 was bold

*ALN* apical lymph node, *IQR* ınterquartile range, *LN* Lymph node, *N/A* non applicable, *NE* neuroendocrine

^a^Fisher’s exact test, ^b^Mann-Whitney U test

**Fig. 2:**
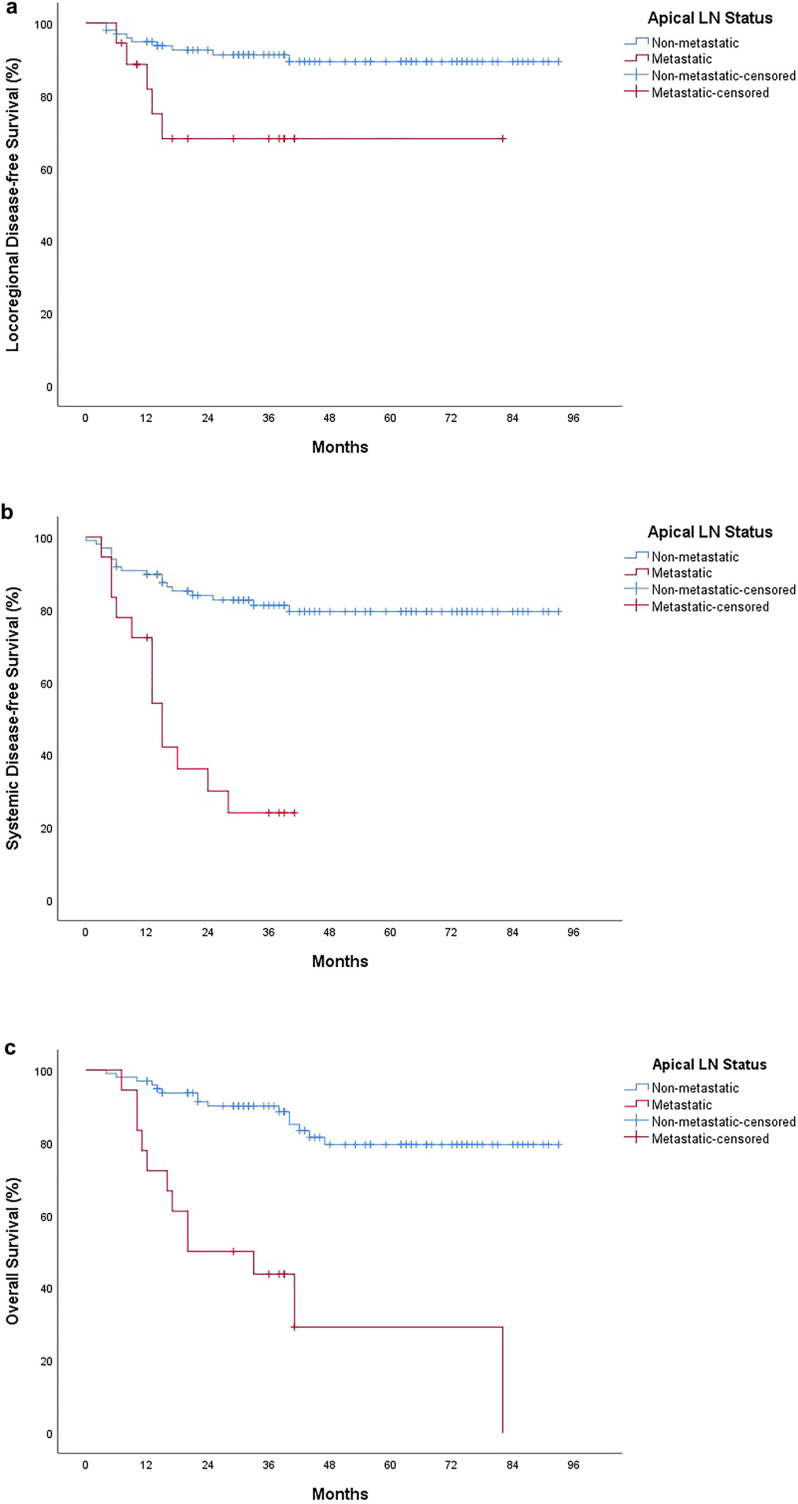
Kaplan–Meier curves depicting survival based on apical lymph node status: **a** Locoregional disease-free survival, **b** Systemic disease-free survival, **c** Overall survival

During a median follow-up of 46 months (IQR: 39–62), 14 local recurrences, 31 systemic recurrences, and 27 cancer-related deaths were recorded. The ALN (+) group had significantly higher rates of local recurrence, systemic recurrence, and cancer-related mortality (*p* = 0.027, *p* < 0.001, and *p* < 0.001, respectively). LDFS, SDFS, and OS were significantly shorter in the ALN (+) group (*p* = 0.011, *p* < 0.001, and *p* < 0.001, respectively). Cox regression analysis was performed to assess the impact of ALN status on survival, independent of pN stage. Although LDFS was shorter in the ALN (+) group, this difference did not reach statistical significance. However, SDFS and OS were significantly shorter in both the ALN (+) and pN2 groups. **(**Tables 4 and 5**, **Fig. 2**).**

**Table 4:** Follow-up and survival times (Postoperative mortalities excluded)

Variables	All patients (N = 115)	ALN (−) Patients (N = 97)	ALN (+) Patients (N = 18)	P-value
Locoregional Recurrence (N, %)				
No	101 (87.8%)	88 (76.5%)	13 (11.3%)	***0.027***^***a***^
Yes	14 (12.2%)	9 (7.8%)	5 (4.3%)	
Locoregional DFS (Months, Median, IQR)	NR (NR-NR)	NR (NR-NR)	NR (15-NR)	***0.011***^***b***^
Systemic Recurrence (N, %)				
No	84 (73%)	79 (68.7%)	5 (4.3%)	** < *0.001***^***a***^
Yes	31 (27%)	18 (15.7%)	13 (11.3%)	
Systemic DFS (Months, Median, IQR)	NR (NR-NR)	NR (NR-NR)	15 (13-NR)	** < *0.001***^***b***^
Vital Status (N, %)				
Alive	88 (76.5%)	82 (71.3%)	6 (5.2%)	** < *0.001***^***a***^
Deceased	27 (23.5%)	15 (13%)	12 (10.4%)	
Overall Survival (Months, Median, IQR)	NR (82-NR)	NR (NR-NR)	27 (16-NR)	** < *0.001***^***b***^

All p-values less than 0.05 was bold

*ALN* Apical lymph node, *DFS* disease-free survival, *IQR* interquartile range, NR NOT reached

^a^Chi-square test, ^b^Log-rank test

**Table 5:** Factors Associated with LDFS, SDFS and OS

Variables	LDFS			SDFS			OS		
Apical LN Status	OR	%95-CI	*P-value*	OR	%95-CI	*P-value*	OR	%95-CI	*P-value*
Non-metastatic	1 (Ref)			1 (Ref)			1 (Ref)		
Metastatic	3.97	0.97–16.29	*0.056*	5.03	1.99–12.72	***0.001***	5.23	1.83–15.26	***0.002***
pN stage									
pN1a	1 (Ref)			1 (Ref)			1 (Ref)		
pN1b	3.78	0.41–34.50	*0.239*	4.75	1.03–21.89	***0.046***	1.98	0.49–7.84	*0.331*
pN1c	3.54	0.22–57.68	*0.375*	1.79	0.16–20.08	*0.635*	1.14	0.12–11.05	*0.913*
pN2a	11.59	1.34–100.5	***0.026***	14.10	3.04–65.42	***0.001***	5.95	1.52–23.38	***0.011***
pN2b	5.19	0.46–58.69	*0.184*	8.74	1.69–45.16	***0.010***	5.21	1.19–22.84	***0.029***

Cox regression analysis. All p-values less than 0.05 was bold

*CI* CONFİDENCE interval, *LDFS* locoregional disease-free survival, *OR* odds ratio, OS overall survival, *Ref* reference, *SDFS* Systemic Disease-free survival

## Discussion

Lymphatic staging in colorectal cancer is a crucial determinant of prognosis, and in the current TNM classification, only the number of metastatic lymph nodes is utilized for staging[4]. In the JCCRC classification, ALN status is defined as a factor that influences the stage [5]. While several studies in the literature indicate that ALN status has prognostic significance and that prognosis can be more accurately determined when combined with the TNM classification, other studies argue that the number of metastatic lymph nodes alone is more useful in determining prognosis [10, 11, 13, 17]. In our study, survival times were significantly shorter in ALN (+) patients, and multivariate analysis revealed that ALN (+) disease is an independent variable associated with poor prognosis. Our findings suggest that ALN status may add prognostic value to TNM staging.

There is heterogeneity in the groups compared in studies examining ALN status, making it difficult to interpret results and establish a clear relationship with ALN status [18, 19]. This has led to the use of techniques such as propensity score matching in some studies to assess the impact of ALN status [13, 20]. Although we did not use such a technique in our study, we found that the ALN (−) and ALN (+) groups were similar in terms of demographic, surgical, and pathological parameters. The only differences were that the pN stage was more advanced and the number of metastatic lymph nodes was higher in the ALN (+) group **(**Table 1, 2 and 3**)**. In multivariate analysis, both pN2 status and ALN (+) were identified as factors that worsen survival. These findings suggest that ALN (+) may hold prognostic significance in addition to TNM staging.

In a study examining the risk of developing distant metastasis during follow-up, Tsai HL et al. [19] reported that ALN metastasis increases the risk of developing systemic metastasis. In our study, the rate of local and systemic recurrences was higher in the ALN (+) group, and LDFS, SDFS, and OS were significantly shorter in this group. Our results support the notion that ALN (+) patients have an increased risk of recurrence, and ALN status may help guide the selection of more aggressive treatment in these patients.

The retrospective nature of our study, the small sample size and non-standardized assesment of ALN are the main limitations of our work. Moreover, the very low representation of right-sided colon cancers in our study limits the generalizability of the findings. Prospective studies with standardized assessment of ALN status will further elucidate its prognostic impact.

## Conclusions

Our findings suggest that ALN metastasis may serve as an independent poor prognostic indicator, in addition to the pN stage. Routine assessment of ALN status may aid in prognostication and guide treatment selection.

## Data Availability

The datasets generated and analyzed during the current study are not publicly available but are available from the corresponding author on reasonable request.
